# Efficacy of 1st-line bismuth-containing quadruple therapies with levofloxacin or clarithromycin for the eradication of *Helicobacter pylori* infection: A 1-week, open-label, randomized trial

**DOI:** 10.1097/MD.0000000000005859

**Published:** 2017-02-17

**Authors:** Jing Su, Xiaoying Zhou, Han Chen, Bo Hao, Weifeng Zhang, Guoxin Zhang

**Affiliations:** a Department of Gastroenterology, First Affiliated Hospital of Nanjing Medical University, Nanjing; b Department of Gastroenterology, XuZhou Central Hospital, Xuzhou, China; c First Clinical Medical College of Nanjing Medical University, Nanjing, China.

**Keywords:** antibiotic resistance, bismuth-containing quadruple therapy, CYP2C19 polymorphism, *H pylori* infection, levofloxacin-based triple therapy

## Abstract

**Background:**

The aim of the present open-label, randomized control trial was to determine the clinical efficacy and safety of two 1-week bismuth-containing quadruple regimens and 1 levofloxacin-based triple regimen for the eradication of *Helicobacter pylori* infection in treatment-naive patients. The influence of susceptibility and host CYP2C19 polymorphisms on the efficacy was also evaluated.

**Methods:**

Eligible patients were randomly to receive esomeprazole and colloidal bismuth pectin along with clarithromycin and amoxicillin (EBCA), esomeprazole and colloidal bismuth pectin along with levofloxacin and amoxicillin (EBLA), or esomeprazole along levofloxacin and amoxicillin (ELA) for 1 week. The primary outcome was the eradication rate in the intention-to-treat (ITT) and per-protocol (PP) analyses.

**Results:**

Overall, 270 patients were randomized. The eradication rates in the above 3 groups were 80.25%, 89.66%, and 81.93% in PP analysis and 72.22%, 86.66%, and 75.56% in ITT analysis, respectively. The eradication rate of EBLA was significantly higher than that of EBCA (*P* = 0.016) in ITT analysis. No significant differences were found among these groups in terms of adverse effects and compliance. The efficacy was significantly affected by levofloxacin resistance for EBLA (*P* = 0.01) and ELA (*P* = 0.04), but not by polymorphisms of CYP2C19 gene for any of the 3 groups.

**Conclusion:**

All 1-week bismuth-containing quadruple therapies and levofloxacin-based triple therapy can obtain an acceptable eradication rate, and levofloxacin-based quadruple regimen exhibits the highest eradication rate. The antibiotic resistant rate of levofloxacin was associated with the eradication rate.

## 1 Introduction

Infection with *Helicobacter pylori* is a substantial public health problem that affects 20% to 50% of people in industrialized nations and up to 80% in less-developed countries.^[1]^ It is an important cause of peptic ulcer disease, gastric carcinoma, and gastric mucosa-associated lymphoid tissue lymphoma.^[2]^ Consequently, eradication of *H pylori* infection has been recommended.^[3]^ Most Consensus Conferences and Clinical Guidelines recommend the triple therapy including a proton pump inhibitor (PPI) and 2 antimicrobial agents as the 1st-line treatment. However, the eradication rates of standard triple therapies have been decreased to less than 80% in many countries recently and the eradication failure is mainly due to poor compliance, antibiotic resistance, and CYP2C19 metabolism.^[4–6]^ Thus, the Maastricht-2012 Consensus Report recommended bismuth-containing quadruple therapy as 1st-line treatment for the eradication of *H pylori* infection in regions with a high clarithromycin resistance rate.^[7]^ As the clarithromycin resistance rate has been reported to be higher than 15% to 20% in China, bismuth-containing quadruple therapy has been strongly recommended as the treatment in China.^[8]^ Although some studies have shown that eradication rate of bismuth-containing quadruple therapy could be improved by extending the duration of treatment from 7 days to 10–14 days,^[9,10]^ other studies have found that there is no significant difference between 7 day and 10–14 day regimens in terms of the efficacy and safety.^[11]^

Levofloxacin-based triple therapy has been shown to be effective as the 2nd-line and 3rd-line rescue regimens for those who have failed to the standard treatment, with an eradication rate ranging from 75% to 90%.^[12]^ Recent studies have also shown that levofloxacin-based triple therapy is effective as the 1st-line treatment, which has also been confirmed in China.^[13,14]^ Success of eradicating *H pylori* infection largely depends on the susceptibility of *H pylori* to the antibiotics used in the regimen.^[15]^ However, most studies did not perform antibiotic susceptibility tests and thus their results cannot be globally generalized due to the varying antibiotic resistance rates among different countries. In addition, host CYP2C19 polymorphisms affect the clinical efficacy of PPIs used in the eradication regimen.^[16]^

Therefore, the present open-label, randomized control trial was designed and conducted to determine the clinical efficacy and safety of two 1-week bismuth-containing quadruple regimens and one 1-week levofloxacin-based triple regimen for the eradication of *H pylori* infection in treatment-naive patients. The influence of susceptibility and host CYP2C19 polymorphisms on the efficacy was also evaluated.

## 2 Methods

### 2.1 Study design and participants

This study was conducted at the First Affiliated Hospital of Nanjing Medical University from March 2013 to October 2013. Consecutive patients with dyspeptic symptoms undergoing upper endoscopy were enrolled. Eligible patients were those aged >18 years who were positive for *H pylori* infection as determined by histology, rapid urease test, serology or ^13^C-urea breath test, and diagnosed with gastritis or duodenal ulcer disease at upper endoscopy. Patients with previous *H pylori* eradication treatment, a history of gastrectomy, gastric ulcer, contraindication or previous allergic reactions to the study drugs, antibiotic administration with the previous 4 weeks, or severe concurrent diseases or malignancy were excluded. Pregnant or lactating women were also excluded.

The primary endpoint of the study was *H pylori* eradication rates in the intention-to-treat (ITT) and per-protocol (PP) analyses. The secondary endpoints were the frequency of adverse events, treatment compliance, antibiotic resistance rate in *H pylori*, and CYP2C19 polymorphisms.

Participants provided written informed consent before entry to the trial. The study protocol was approved by the Institutional Review Board of the First Affiliated Hospital of Nanjing Medical University. This trial was registered with Chinese Clinical Trial Registry, Number: TRC13003256.

### 2.2 Randomization and treatment procedures

Patients who met the inclusion criteria were randomized (1:1:1) assigned to receive one of the following regimens: colloidal bismuth pectin 200 mg, clarithromycin 500 mg, amoxicillin 1000 mg, and esomeprazole 20 mg, all twice daily (EBCA); colloidal bismuth pectin 200 mg twice daily, levofloxacin 500 mg once daily, amoxicillin 1000 mg twice daily, and esomeprazole 20 mg twice daily (EBLA); and levofloxacin 500 mg once daily, amoxicillin 1000 mg twice daily, and esomeprazole 20 mg twice daily (ELA). All drugs were taken for 7 days. For patients with duodenal ulcers, additional 2 weeks’ esomeprazole 20 mg twice daily was needed after the 1-week eradication regimens. Randomization was carried out using a permuted block with a size of 6, and the random number sequence was generated by the computer and was concealed in an opaque envelope until the intervention was assigned.

*H pylori* infectious status was reassessed by ^13^C-UBT at least 4 weeks after the completion of the treatment. Patients were asked to stop PPIs or H_2_ blocker for at least 4 weeks before follow-up testing. The urea kit (which contained 75 mg ^13^C-urea) was dissolved in water and mixed with orange juice. Baseline and 30 minutes breath samples were assayed with a mass spectrometer (KYKY Technology Development Ltd, Beijing, China). Positive results were defined when the Δ value was 4 units or higher.

Patients were asked to record any adverse events occurred during therapy in a diary, which included diarrhea, taste disturbance, nausea, bloating, loss of appetite, vomiting, abdominal pain, constipation, headache, and skin rash. Serious adverse events were defined as those symptoms patients considered as disrupting their daily life and must stop the treatment. In the 1st week after the completion of therapy, a telephone interview was also arranged to assess the adverse events and compliance. Compliance was recorded as low when less than 80% of pills were taken.

### 2.3 CYP2C19 genotyping

CYP2C19 genotyping was performed for each of the treated patients. Briefly, blood sample (2 mL) was collected into a vacuum tube with EDTA. DNA was isolated from the blood using the RelaxGene Blood DNA System (Tiangen Biotech Co, Ltd, Beijing, China). Then, PCR restriction fragment-length polymorphism (RFLP)-based analysis was carried out with following primers designed by the Hua Gene Biotech Company (Beijing, China). The forward and reverse primers of mutation1 (m1) were 5′-CAACCAGAGCTTGGCATATTG-3′ and 5′-CACAAATACGCAAGCAGTCAC-3′, respectively. The forward and reverse primers of mutation 2 (m2) were 5′-CACCCTGTGATCCCACTTTC-3′ and 5′-CTAATGGGCTTAGAAGCCTG-3′, respectively. The amplified PCR products, which were 301 and 376 bp gene fragments for m1 and m2, respectively, were digested with enzyme, *Sma*I for m1 and *BamH*I for m2 (New England Biolabs, Beverly, MA). Then, the digested PCR products were analyzed on a 1.5% agarose gel and stained with ethidium bromide. Because CYP2C19 m1 lacks *Sma*I site and CYP2C19 m2 lacks *BamH*I site, the mutant alleles are resistant to endonuclease digestion. Meantime, we chose 70 samples for direct sequencing (Hua Gene Biotech Company) to verify the accuracy of PCR-RFLP. Studied patients were categorized into 3 groups, based on the existence of m1 or m2 on CYP2C19 genotyping: homozygous extensive metabolizers (wild type, wt/wt); heterozygous extensive metabolizers (wt/m1 or wt/m2), and poor metabolizers (PM) (m1/m1, m1/m2, or m2/m2).

### 2.4 Antibiotic susceptibility test

Antibiotic susceptibility in *H pylori* was tested by genotyping 23S rRNA for clarithromycin and *gyrA* for levofloxacin. Fecal samples were collected from the patients prior to the treatment. Genomic DNA from stool specimens was extracted using the QIAamp DNA Stool Mini Kit (QIAGEN, China). The *H pylori* 23S rRNA gene fragment was amplified by nest PCR with following primers described by Noguchi.^[14]^ The forward and reverse primers were 5′-GGTCTCAGCAAAGAGTCCCT-3′ and 5′-CCCACCAAGCATTGTCCT-3′, respectively, in the 1st-round, and 5′-AGGATGCGTCAGTCGCAAGAT-3′ and 5′-CCTGTGGATAACACAGGCCAGT-3′, respectively, in the 2nd-round. The PCR products were then purified and digested with restriction endonucleases *Bsa*I, *Bbs*I, and *BceA*I, and the fragments were cut off when the mutation was determined as A2143G, A2142G, or A2142C, respectively. The PCR products were also sent to Shanghai Sangon Biotech Corporation (Shang, China) to conduct the DNA sequencing. Similarly, the *gyrA* fragment was amplified by nest PCR with the following primers: forward and reverse: 5′-GATCATAGGGCGCTTTACC-3′ and 5′-TTCCCACTGGTGGGATCAT-3′, respectively, in the 1st-round, and 5′-GCTTAAAGCCCGTGCATAGG-3′ and 5′-GACGCTTTAGCGCATGTCT-3′, respectively, in the 2nd-round. The PCR products were also sent for DNA sequencing. Nucleotide sequences were aligned and analyzed using the DNA Star software package. The gene sequence of *H pylori* strain 26695 (ATCC) was used as the quality control strain.

### 2.5 Sample size estimation and statistical analysis

Previous studies have suggested that the eradication rates of EBCA would be about 80%.^[9]^ Consequently, our initial estimation of the sample size was at least 73 individuals in each group to determine the 10% difference in the clinical efficacy, considering a power of 90% and a 0.025 two-sided type 1 error, assuming 10% loss to follow-up. Finally, we decided to increase our sample size to 90 individuals each group to allow a more drop-out rate.

ITT and PP analyses were performed to calculate the eradication rate. The ITT analysis included all randomized patients. Individuals who did not taking at least 80% of the drugs, or with unknown posttreatment *H pylori* status were excluded from the PP analysis. Chi-square test was used to determine the difference among and between the three groups; odds ratio and 95% confidence interval (CI) were calculated where appropriate. Patients who did not return for a follow-up ^13^C-UBT were defined as being lost to follow-up. To assess factors affecting eradication rates, univariate analyses and multiple logistic regression analyses with the following predictors of interest: age, sex, body-mass index (BMI), clarithromycin resistance, levofloxacin resistance, CYP2C19 genotype, peptic ulcer disease, smoking, and drinking were performed. SPSS Software (SPSS Inc. Chicago, IL) was used for statistical analyses. The significance level was set at a *P* value of less than 0.05.

## 3 Results

### 3.1 Patient characteristics

In total, 1200 dyspepsia patients were screened and 464 (38.7%) patients were positive for *H pylori* infection. Of these 464 patients, 171 patients were previously treated with anti-*H pylori* regimens and 23 refused to participate in the study. Thus, 270 patients were recruited in the trial. There were no significant differences in the basic characteristics, including age, sex, cigarette smoking, alcohol drinking, BMI, and endoscopic diagnosis among the 3 groups before 1st-line treatment (Table 1).

**Table 1:**
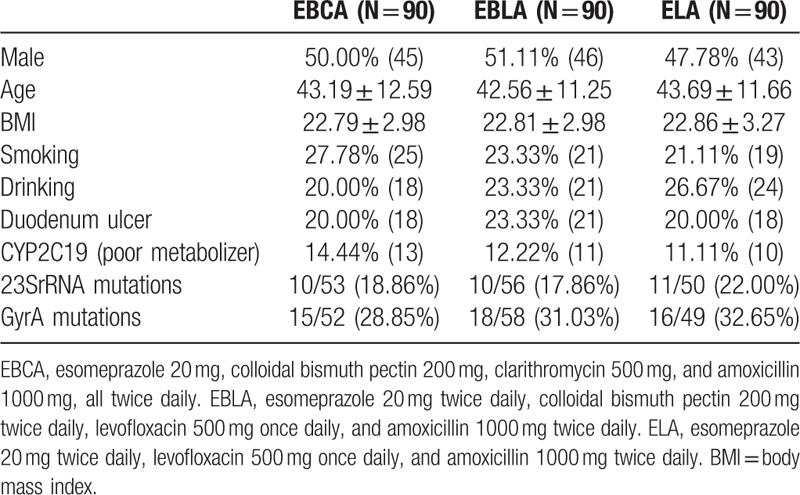
Baseline characteristics of patients randomized in the 3 groups.

### 3.2 *H pylori* eradication rates

Overall, eradication rates were 78.2% (211/270, 95% CI: 76.2%–87.6%) and 84.1% (227/270, 95% CI: 77.6%–88.8%), respectively, in the ITT and PP analyses. The corresponding rates in groups EBCA, EBLA, and ELA were 72.2% (65/90), 86.7% (78/90), and 75.6% (68/90) (χ^2^ = 6.029, *P* = 0.049), respectively, in the ITT analysis, and 80.3% (65/81), 89.7% (78/87), and 81.93% (68/83) (χ^2^ = 3.194, *P* = 0.203), respectively, in the PP analysis (Table 2). The eradication rate was significant greater in group EBLA than in group EBCA (χ^2^ = 5.749, *P* = 0.016) in ITT analysis. There was no significant difference in eradication rates between any 2 of the 3 groups in PP analysis.

**Table 2:**
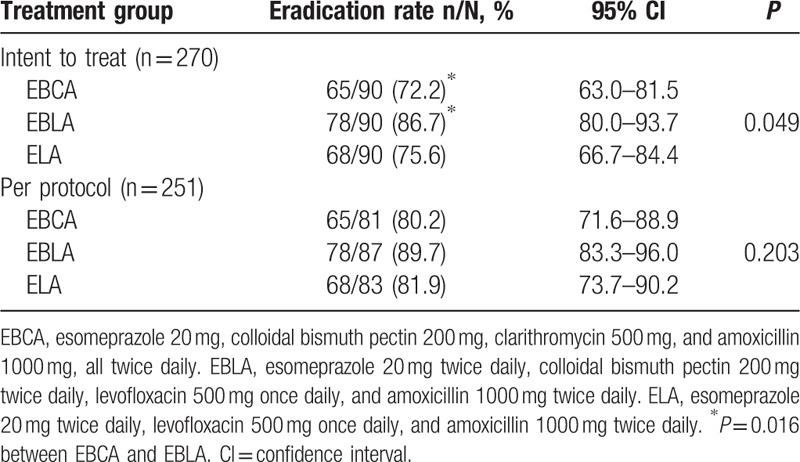
*Helicobacter pylori* eradication rates in intention-to-treat and per-protocol analyses.

### 3.3 Adverse effects and compliance

Adverse events are shown in Table 3. Five patients discontinued drugs because of serious allergic reaction (n = 4) or mental disorders (n = 1). The occurrence of each symptom was not significantly different among the 3 groups. In addition, there was no significant difference in the compliance among the 3 groups; the rates were 96.7% (87/90), 98.9% (89/90), and 98.9% (89/90) in group EBCA, EBLA, and ELA, respectively.

**Table 3:**
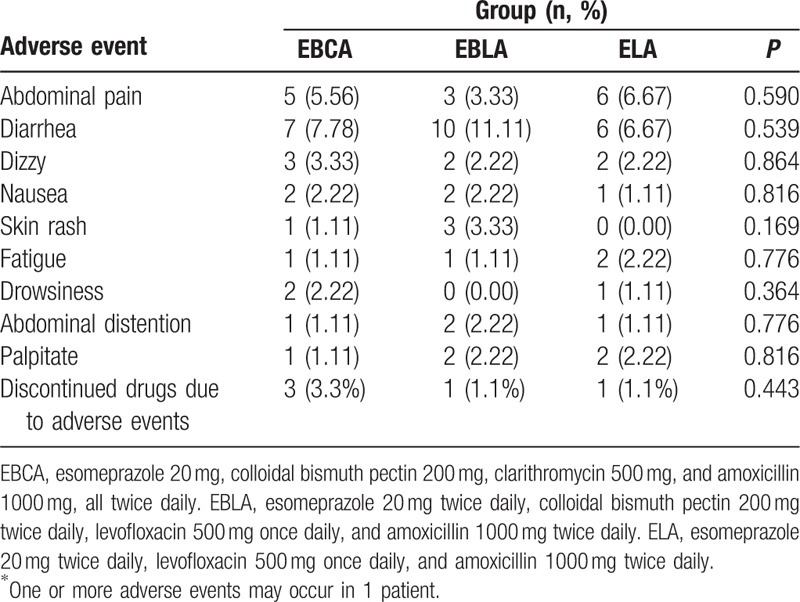
Adverse events among 3 groups in intention-to-treat analysis.

### 3.4 Effect of CYP2C19 polymorphism on efficacy

CYP2C19 genotyping was successfully performed on blood samples from all patients. Based on genotyping, 113 (41.85%), 123 (45.56%), and 34 (12.59%) patients were classified as homozygous extensive metabolizers, heterozygous extensive metabolizers, and poor metabolizers, respectively. There were no significant differences in the distribution of CYP2C19 genotypes among the 3 groups. ITT and PP analyses showed that the efficacy of the 3 therapies was not affected by host CYP2C19 polymorphism (Table 4).

**Table 4:**
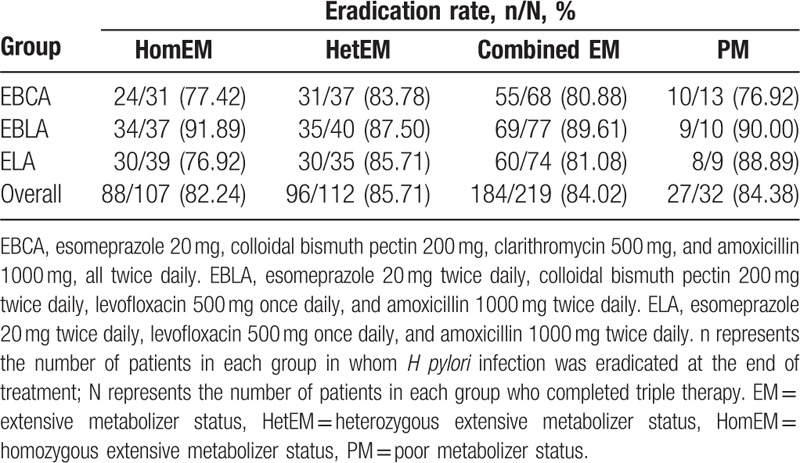
Effects of the CYP4502C19 genotype on rates of *Helicobacter pylori* eradication, by treatment regimen, in the per-protocol population.

### 3.5 Effect of antibiotic resistance on efficacy

Antibiotic susceptibility testing results were available in 189 patients, 30 for clarithromycin alone, 30 for levofloxacin alone, and 129 for both. Overall, the rates of resistance to clarithromycin and levofloxacin were 19.05% (31/159) and 30.82% (49/159), respectively; 1 patient had *H pylori* resistant to both clarithromycin and levofloxacin. During the treatment, 19 patients (9, 3, and 7 from groups EBCA, EBLA, and ELA, respectively) were lost to follow-up.

Among patients in EBCA regimen, clarithromycin resistance was associated with a lower eradication rate (83.7% vs 55.6%, χ^2^ = 3.54, *P* = 0.080). Moreover, the eradication rate was significantly affected by levofloxacin resistance in EBLA (92.3% vs 61.1%, χ^2^ = 8.29, *P* = 0.010) or ELA (90.9% vs 66.7%, χ^2^ = 5.8, *P* = 0.040), as shown in Table 5.

**Table 5:**
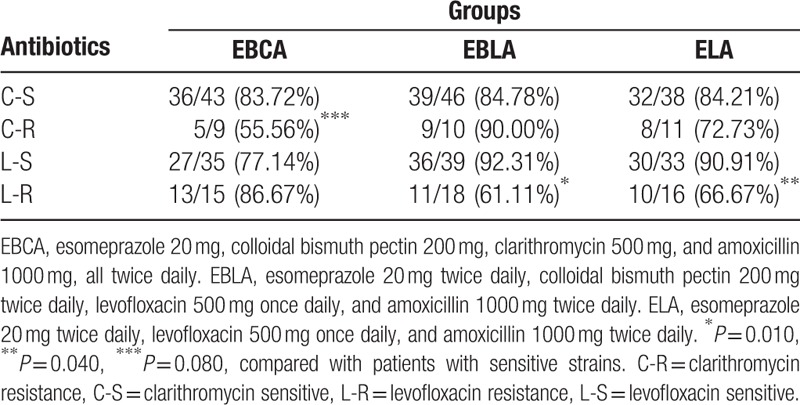
Effect of the antibiotic resistance on the clinical efficacy of the 3 regimens.

### 3.6 Other potential factors affecting efficacy

After excluding 19 patients who lost to follow-up, we analyzed other potential factors which might affect the eradication rates using univariate and multivariate logistic regression analyses (Table 6). BMI was found to be an independent factor affecting *H pylori* eradication rate; the higher the BMI index, the lower the success rate of eradication. However, other factors, including sex, age, smoking, drinking, and CYP2C19 polymorphism, did not affect the eradication rate.

**Table 6:**
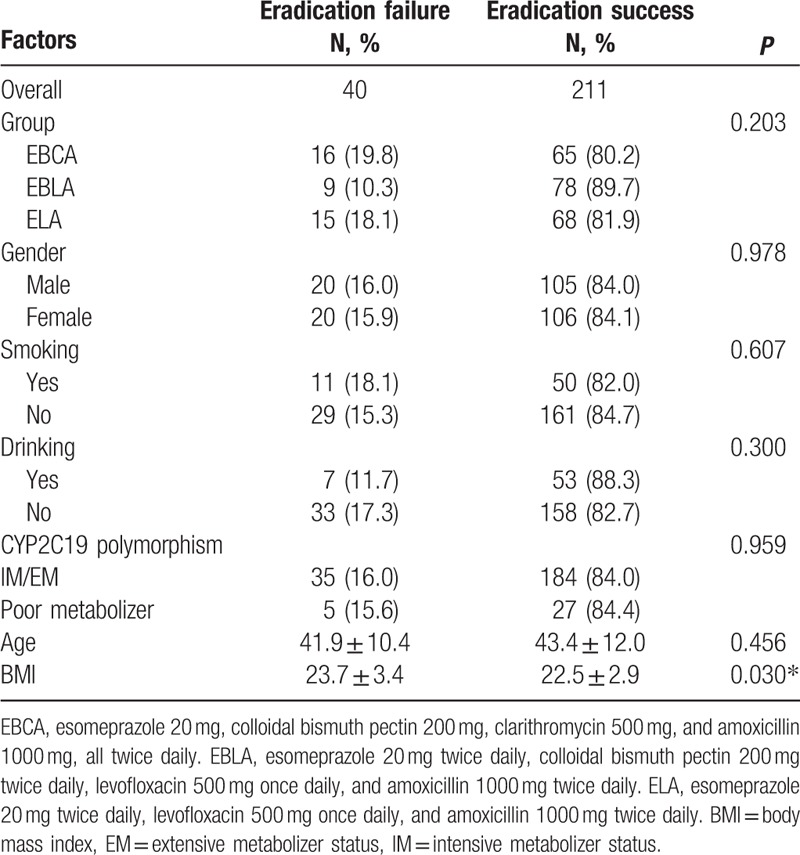
Factors affecting eradication rates as determined in univariate analyses.

## 4 Discussion

As the antibiotic resistance rates, especially to metronidazole and clarithromycin, have been increasing over the decades, the standard triple therapy could not reach the acceptable eradication rate of 80% in most studies.^[16,17]^ In response to the rising resistance rates, new therapies including quadruple therapy (eg, bismuth-containing quadruple therapy or concomitant therapy) and sequential therapy (eg, 5-day esomeprazole and amoxicillin followed by 5-day esomeprazole, clarithromycin, and tinidazole) have emerged as alternatives to the standard therapy.^[18]^ Concomitant therapy including more than 2 antibiotics is not recommended to be the 1st-line regimen considering the antibiotic adverse events and resistance. Sequential therapy remains a hot topic in *H pylori* eradication, showing generally superiority over classic triple therapy. However, the regimen is relatively complex requiring the patient to switch from a dual to a triple therapy at middle point.^[19]^ Moreover, some studies from China did not show any advantages of sequential therapy over the standard triple therapy.^[8]^

Bismuth has an established history in the treatment of *H pylori*, which has a synergistic effect with antibiotics and decreases the bacterial load to increase the eradication rate. Importantly, there is no resistance to bismuth in *H pylori*, which makes bismuth a preferred antimicrobial agent for *H pylori* eradication.^[20,21]^ Bismuth-containing quadruple therapy has usually been used as an optimal rescue regimen in guidelines and achieved a high eradication rate in the past.^[22–24]^ In Maastricht IV Consensus Report, it is reported that in areas of high clarithromycin resistance, bismuth-containing quadruple treatment is recommended for 1st-line empirical treatment.^[7]^ A recent study showed an eradication rate of 90.7% for a 14-day bismuth-containing quadruple therapy as 1st-line therapy in Turkey.^[25]^ In the present study, the eradication rate of 2 bismuth-based quadruple regimens was almost 85%, although it is lower than the previously reported which may be due to the shorter treatment duration. Given the wide indications for *H pylori* eradication and the importance of choosing the optimum duration of therapy, it is necessary to further determine whether longer treatment duration would be beneficial to obtain better eradication rate. Although the 14-day regimen has been generally preferred for the 2nd-line or subsequent treatment, studies evaluating the duration of bismuth-containing quadruple therapy as 1st-line treatment are rare. A recent study found that the 14-day bismuth-containing quadruple regimen led to a significant increase of *H pylori* eradication compared with 7-day regimen in the ITT analysis (93.7% vs 80.0%; *P* = 0.01).^[10]^ A recent Cochrane review of 6 randomized controlled trials and 4763 patients assessed the eradication rates of different bismuth-containing quadruple regimens with different durations and found no significant difference among 7, 10, and 14 days.^[26]^ Thus, considering the assumption that prolonged treatment duration is more expensive, and may lead to increased drug-related adverse reactions and poor compliance, we adapted 7-day duration in the present study and showed that 7-day bismuth-containing quadruple regimens with levofloxacin achieved an eradication rate of 86.7% as 1st-line treatment. In addition, 7-day bismuth-containing quadruple regimens with clarithromycin and levofloxacin-based triple regimen also achieved an eradication rate above the accepted threshold of 80%.^[27]^ Moreover, all these 3 regimens were associated with relatively low rates of adverse events.

Levofloxacin, a broad-spectrum fluoroquinolone agent, has been postulated as an efficient alternative to clarithromycin for triple, quadruple, or sequential regimens.^[28]^ When used in a triple 1st-line regimen, it has been evaluated to achieve higher eradication rates than clarithromycin-containing triple therapy.^[29]^ Two studies from China demonstrated that the eradication rates of a 7-day levofloxacin-based triple treatment ranged from 80.9% to 86.7%.^[27,30]^ A randomized, blind, multicenter study from Middle East showed that 7-day levofloxacin-based triple treatment was superior to the standard triple therapy in terms of the eradication rate (84.7% vs 74.7%).^[31]^ In the present study, the overall eradication rate of the 3 regimens was 84%, which was similar to previously reported. Moreover, the combined eradication rate of the 2 levofloxacin-based regimens was 86%, which is well above the acceptable rate.

Antibiotic resistance is the main factor that contributes to the eradication failure of *H pylori* infection. A large-scale multicenter European study revealed resistance rates of 17.5% for clarithromycin, 14.1% for levofloxacin, and 34.9% for metronidazole, with a significant association between outpatient antibiotic use and the proportion of antibiotic resistance.^[32]^ In China, resistance rates to clarithromycin, metronidazole, and levofloxacin were 21.5, 95.4, and 20.6%, respectively,^[33]^ which are similar to our study. There is almost no resistance to amoxicillin in *H pylori* strains in China. In the present study, we found that *H pylori* resistance to clarithromycin or levofloxacin affected the efficacy of 3 therapy regimens in which clarithromycin or levofloxacin was used. The high prevalence of clarithromycin resistance (19.5%) and levofloxacin resistance (30.8%) in the present study accounted for the relevantly low eradication rate of all the 3 treatments. In addition, in the present study, the levofloxacin-based quadruple and triple regimens appear more efficacious than the clarithromycin-containing quadruple regimen as 1st-line treatment after excluding the factor of antibiotic resistance; the eradication rates were 83.7% in EBCA, and 92.3% and 90.9% in groups EBLA and ELA).

The strength of our study included comparison of 3 different 1st-line treatments, extensive analysis of factors that might affect treatment efficacy. In addition, we proved that 1-week bismuth-containing quadruple therapy and levofloxacin triple therapy could achieve an acceptable eradication. Nevertheless, there are also some limitations in this study. First, antibiotic susceptibility data were available in only 59% of patients, which might raise the possibility of selection bias. This was because not all patients could provide fecals since they came for and also related to the fact that the culture rate of *H pylori* is less than perfect. Second, we did not research their efficacy when they were used as rescue regimens. Because of our small samples, we could not enroll enough patients in 2nd ground treatments. Third, our study was open label. Although we recorded no substantial difference between the baseline characteristics of the ITT and PP study population, patients who are lost to follow-up or noncompliant might be as a direct result of their treatment allocation so that the PP population had higher eradication rates for each group.

In conclusion, 1-week bismuth-containing quadruple regimen with levofloxacin or clarithromycin and levofloxacin-based triple regimen achieved an acceptable eradication rate for *H pylori* infection, with the bismuth-containing quadruple regimen with levofloxacin exhibiting the highest eradication rate. The resistance to clarithromycin or levofloxacin is associated with decreased efficacy. In addition, BMI, not sex, age, smoking, drinking, and CYP2C19 polymorphism, is an independent factor affecting the efficacy of the regimens (TRC13003256).

## Acknowledgments

The authors thank National Natural Science Foundation of China (No. 81270476 and 81470830) and the Priority Academic Program Development of Jiangsu Higher Education Institutions (JX10231801) for the support.
